# Deriving Animal Behaviour from High-Frequency GPS: Tracking Cows in Open and Forested Habitat

**DOI:** 10.1371/journal.pone.0129030

**Published:** 2015-06-24

**Authors:** Nelleke de Weerd, Frank van Langevelde, Herman van Oeveren, Bart A. Nolet, Andrea Kölzsch, Herbert H. T. Prins, W. Fred de Boer

**Affiliations:** 1 Resource Ecology Group, Wageningen University, Wageningen, the Netherlands; 2 Department of Animal Ecology and Project Group Movement Ecology, Netherlands Institute of Ecology (NIOO-KNAW), Wageningen, the Netherlands; The Australian National University, AUSTRALIA

## Abstract

The increasing spatiotemporal accuracy of Global Navigation Satellite Systems (GNSS) tracking systems opens the possibility to infer animal behaviour from tracking data. We studied the relationship between high-frequency GNSS data and behaviour, aimed at developing an easily interpretable classification method to infer behaviour from location data. Behavioural observations were carried out during tracking of cows (*Bos Taurus*) fitted with high-frequency GPS (Global Positioning System) receivers. Data were obtained in an open field and forested area, and movement metrics were calculated for 1 min, 12 s and 2 s intervals. We observed four behaviour types (Foraging, Lying, Standing and Walking). We subsequently used Classification and Regression Trees to classify the simultaneously obtained GPS data as these behaviour types, based on distances and turning angles between fixes. GPS data with a 1 min interval from the open field was classified correctly for more than 70% of the samples. Data from the 12 s and 2 s interval could not be classified successfully, emphasizing that the interval should be long enough for the behaviour to be defined by its characteristic movement metrics. Data obtained in the forested area were classified with a lower accuracy (57%) than the data from the open field, due to a larger positional error of GPS locations and differences in behavioural performance influenced by the habitat type. This demonstrates the importance of understanding the relationship between behaviour and movement metrics, derived from GNSS fixes at different frequencies and in different habitats, in order to successfully infer behaviour. When spatially accurate location data can be obtained, behaviour can be inferred from high-frequency GNSS fixes by calculating simple movement metrics and using easily interpretable decision trees. This allows for the combined study of animal behaviour and habitat use based on location data, and might make it possible to detect deviations in behaviour at the individual level.

## Introduction

Global Navigation Satellite Systems (GNSS) allow ecologists to gain information on movement, habitat use and home ranges of animals [1,2]. While visual observations are often restricted, GNSS tracking devices allow the near-continuous study of animals [3]. The positional accuracy and temporal frequency of measured locations has increased with the development of new GNSS tracking systems [4,5]. This provides more detailed information on movement patterns and habitat use, and additionally opens the possibility to derive the animals’ behaviour from tracking data [6–9].

An increasing number of studies have derived ‘behavioural modes’ from movement data [6, 10, 11]. However, only a few have done this at time intervals smaller than several hours or minutes [3, 12, 13], and even fewer used simultaneous observations to link tracking data to actual behaviour, and carried out an appropriate testing and validation procedure [3, 14]. Many of these studies incorporated tracking data into complex movement models in order to calculate the pattern of behaviour during the day [15] or to predict movements [10]. Recently, tracking studies often incorporate accelerometer data in their analyses [3, 16], without using the increasing possibilities of GNSS tracking.

From GNSS locations, movement metrics like the distance travelled, speed, heading and turning angles between fixes can be derived, which can be used to infer different types of behaviour [3, 5, 10, 11]. For instance, Guo et al. [12] fitted six cows with GPS (Global Positioning System) tracking devices, and calculated the directional speed as well as angular speed (rotation rate) between fixes. Based on visual observations of animal activities, Guo et al. [12] subsequently set various thresholds for these variables representing different modes of behaviour (foraging: large turning angles and low speeds; walking: small turning angles and high speeds; resting: small turning angles and low speeds). Similarly, Fryxell et al. [11] showed that the turning angle distribution can describe behaviour of elk (*Cervus elaphus)*, with distributions being more variable and movements being less direct while foraging than while searching.

The higher spatiotemporal accuracy of tracking data is expected to increase the precision of the movement metrics calculations, enabling a more accurate inference of the behaviour an animal is performing. Linking this behaviour to the location and thus the habitat type the animal resides in, increases the understanding of foraging behaviour and resource use [1, 11, 17], providing a basis for management decisions [18]. This is not only of interest to wildlife biologists and conservation managers, but also to farmers who can apply information on the spatial location of their grazing livestock to improve use of forage availability [12, 14, 15, 17]. A higher spatiotemporal accuracy will furthermore enable the quantification of individual differences in behaviour. This possibly provides researchers, managers or farmers with the ability to deduce deviant behaviour of individuals (e.g., being ill, lame or in oestrus) from tracking data. This may allow for the mitigation of disease transmission (between wildlife and livestock) in an early stage, or offer a method to increase welfare and production of livestock [19].

Tracking of wildlife in its natural surroundings is still found to be subject to impaired reception of the GNSS signal [4, 20, 21], especially by forest cover. Previous studies have shown that both forest type and structure influence position acquisition rate and positional accuracy [22,23]. GPS performance decreases when the signal is blocked by trees, and fix success rate decreases and positional error increases under forest canopies [23–26].

Because the true potential of the improvements in tracking devices, for a wide range of end-users, is still poorly investigated [8, 27], we studied the accuracy of a behavioural classification based on GNSS tracking data. We analysed the relationship between high-frequency GNSS data in terms of the animal’s movement metrics and its behaviour. We show how this relationship can be applied to infer behaviour from GNSS data. In contrast to studies that incorporated tracking data into complex movement models, we developed an easily interpretable classification method to infer behaviour from every interval between two location fixes. We analysed cow (*Bos taurus*) movement patterns measured from GPS location fixes, and tested whether GPS fixes obtained at ever-smaller time intervals improve the classification accuracy of cow behaviour. Forest cover was expected to increase the errors in GPS locations compared to an open field situation, and we therefore expected a lower classification success rate of the GPS data in terms of cow behaviour in a forested environment than in an open field.

## Materials and Methods

### Ethics statement

The study was carried out on private land, and permission was granted by the land owners.

The study did not involve endangered or protected species and did not require the approval of an animal ethics committee or specific permissions with regard to locations or activities as the research activities were non-invasive, did not cause any inconvenience to the animals, and the GPS sensors were fitted indoors when the cows were stalled.

### Data collection

We conducted our work in an open field with dairy cows (breed: Holstein Friesian), but also with free-ranging cows (Blaarkop) in an area that is partly forested, to assess the effect of tree cover. The studies in the open field and the forest were carried out under similar weather conditions. The open field study was performed on a farm with dairy cows near Wageningen, the Netherlands (52°01’ N, 5°62’ E). Observations were done on a rectangular open field where the cows grazed between 08:00 and 17:00 hours; during this time the cows could drink freely at a watering point (S1 File). Nine cows were fitted with an EGNOS-enabled Lotek GPS6000M sensor (Biotrack Ltd., Wareham, Dorset, UK) in a collar around their necks. The sensors were subjected to exploratory tests to determine the performance of the sensors in terms of positional accuracy. All sensors were mounted on fixed poles during one day with weather conditions similar to the conditions when the cows were observed. One collar was mounted on a fixed pole during the open field study as a reference, which showed an average positional error of 2.3 m (against the location obtained from a Leica DGPS, 1200+, RTK system). The tracking of the cows was carried out during 10 days, spread over the period 10–26 July 2012, during which the sensors were set to record locations at 1 min intervals. The cows followed a similar grazing and resting pattern every day, and in order to capture all types of behaviour in the 10 day observation period, the sensors were programmed in advance to include a 2 s interval logging period for each cow at a different time each day. Every cow was observed continuously during 15 min in the morning and 15 min in the afternoon. These visual observations of the behaviour were carried out from 07:00–14:00 by observing the cows in a random order. Additionally, each day the cow whose sensor was programmed to give a position fix every 2 s (the smallest time interval technically possible) was observed for one 15 min period. The behaviour of the cows during the observation periods was classified using an ethogram (Table 1), using mutually exclusive behavioural classes. The start and end of each behaviour within the 15 min period was recorded on a Psion Workabout with Pocket Observer 2 software and The Observer XT 10 software (Noldus IT, Wageningen, The Netherlands). Observations were carried out by two individuals (HvO and NdW), who part of the time observed cows simultaneously to account for inter-observer differences, reducing the bias of behaviour classification. Cows were stalled during the night and no observations were carried out at nighttime, due to the reduced performance of the dominant behaviour types.

**Table 1:** Different types of behaviour and the definitions used to record them during observations.

Behavior	Description	Open field			Forest		
		1 min interval	2 s interval	12 s interval	1 min interval	2 s interval	12 s interval
*Walking*	Movement from one location to another without holding head at ground level	68	297	72	17	307	53
*Foraging*	Grazing or browsing taking frequent bites of forage, without lifting head up	976	769	394	552	727	354
*Standing*	Standing still, no movement to another location	68	90	21	171	45	57
*Lying*	Cow is lying down	104	33	12	699	63	141
*Drinking*	Drinking water	-	-	-	-	-	-
*Grooming*	Cleaning or scratching itself	-	-	-	-	-	-
*Social*	Interaction with other cows (e.g., grooming, mounting)	-	-	-	-	-	-
*Dry Forage*	Consuming silage left by the farmer	-	-	-	-	-	-

The number of intervals *(n)* that contained only one type of behaviour between fixes is provided for the four dominant types of behaviour for the dataset of the 1 min, 12s and 2 s interval in both the open field and forest habitat.

The forest study was performed at the “Renkumse Heide” (S1 File) near Wageningen (52°01’ N, 5°75’ E), where eight free-ranging cows were fitted with tracking sensors. The sensors were programmed in the same way as during the open field study and the behaviour was recorded during visual observations based on the same ethogram (Table 1). In addition, two sensors were placed on a fixed location as a reference, one inside and one outside of the forest.

### Data processing and analysis

In order to analyse how distance and turning angle derived from GPS data related to the behaviour performed by the cows, only those GPS data were used during which visual observations were carried out simultaneously. The duration of observed behaviours ranged from several seconds to maximally 15 minutes. The data were analysed using intervals of 1 min, 12 s or 2 s. We used the 2 s dataset to analyse GPS data at a 12 s interval, by selecting the locations every 12 s. Next, in order to relate the observed behaviour to movement metrics, the observation data was coupled with the GPS data. For every GPS interval a distance and a turning angle were calculated and these were related to the behaviour that was observed within that same interval. From the GPS data intervals were selected during which only one type of behaviour occurred (e.g., for the 1 min interval: one full minute of Walking) between every two subsequent fixes. The behaviour that was observed between two subsequent fixes was coupled to the distance covered between these two fixes and to the turning angle at the first fix. Distances were calculated in meters and turning angles in degrees using the adehabitatLT package in R [28].

For further analysis, fixes were selected during which the cows (based on visual observations) were Foraging, Lying, Standing or Walking, being the four most dominant behaviours. Subsequently, all GPS data with simultaneous observations was used, in order to examine the classification success for 1 min intervals in which multiple types of behaviour occurred. This was done in order to approximate a typical field situation in which data is collected without knowledge of a change in behaviour between fixes. We aimed at classifying this GPS data as the behaviour that was most dominant within these intervals.

For the forest study the same data selection was carried out, using 1 min, 12 s and 2 s time intervals. ‘Time of day’ was incorporated in the classification as an auxiliary variable. For this, the visual observations were divided into categories of half an hour and a full hour (e.g., 08:00–08:30 or 08:00–09:00). Per time category the average percentage of time allocated to the different behaviours was calculated. This was then used to explore whether the recurrence of a particular behaviour in time can be used as an extra predictor variable to improve the correct classification of the GPS data [29] (S2 File).

Because sample sizes between the behavioural classes differed largely and movement metrics of behavioural classes did not have equal variances, we used a permutation ANOVA from the lmPerm package in R [30] to test for differences in movement distances and turning angles between the different behaviour types. The conservative permutation ANOVA was not able to incorporate the repeated structure of the data, but because multiple measurements of the same cow were collected over various days, we assume that the repeated measurements do not largely bias the ANOVA. The permutation ANOVA was also carried out for all pairwise comparisons as a post-hoc test [31], using a Bonferroni correction [32].

### Decision tree building

Decision trees were constructed using the Classification and Regression Tree (CART) method [16, 33]. These decision trees were used to classify the GPS data on the basis of differences in movement metrics between behaviour types. With a decision tree a set of decision rules is created. Starting at node 0, the input data is split up, based on one of the input variables (distance and turning angle) by a decision rule. After this split, a new decision rule can further split up the data, until a terminal node is created which predicts the class (in this case behaviour). The advantage of the CART method is that the decision rules are easy to interpret and directly applicable for classification of new data [3, 16].

For these analyses half of the data of each time interval (1 min, 12 s or 2 s) and habitat (open field or forest) was used to create the model (training sample), whereas the other half was set aside for validation afterwards. The optimal decision tree must contain criteria that classify the data as accurately as possible, but these criteria should not be too complex because of the risk of ‘over-fitting’ the data [33, 34]. First a decision tree was created as a reference, which was allowed to split and add nodes without defined limitations. The optimal size for the decision tree was then decided upon using *k*-fold cross-validation, using *k* = 10 [32], in which the training dataset is divided into *k* folds of which one fold is used each time as a training sample and the other *k-*1 folds are used as test samples [35, 33]. This gives a decision tree with a risk estimate as a measure for the chances of misclassification. Risk estimates were averaged over 10 runs and then plotted against the number of nodes, which was varied as a measure of tree complexity [36]. The number of nodes that was associated with the lowest risk estimate, but still able to differentiate between all types of behaviour given as input, was then chosen for the optimum tree size and the reference tree was subsequently pruned to approach the same number of nodes. The resulting tree was used as a model and validated with the other half of the dataset.

## Results

### Behavioural observations

Of the nine cows wearing GPS sensors in the open field, eight were successfully tracked during the complete 10 day observation period. One of these eight sensors failed to log during the third day due to an error in the programmed schedule. Except for this error, the sensors succeeded in requiring a location fix 99.3% of the time. In the forest, all eight cows wearing sensors were successfully tracked during both the 1 min and 2 s time intervals over the complete 10 day observation period. The sample sizes from both habitats and all three time intervals that were used for further analysis are shown in Table 1 for the behaviours Walking, Foraging, Lying and Standing.

### Open field

All sensors had a 100% fix success rate (there were no missing fixes) and the average positional error of the sensors in the open field was 2.1 m (SD = 0.2 m). For the 1 min, 12 s and 2 s time interval in the open field, respectively, 2565, 651 and 1387 intervals between two subsequent fixes could be linked to simultaneous observations of which 1316, 524 and 1189 intervals contained only one type of behaviour.

#### 1 min interval

**Fig 1:**
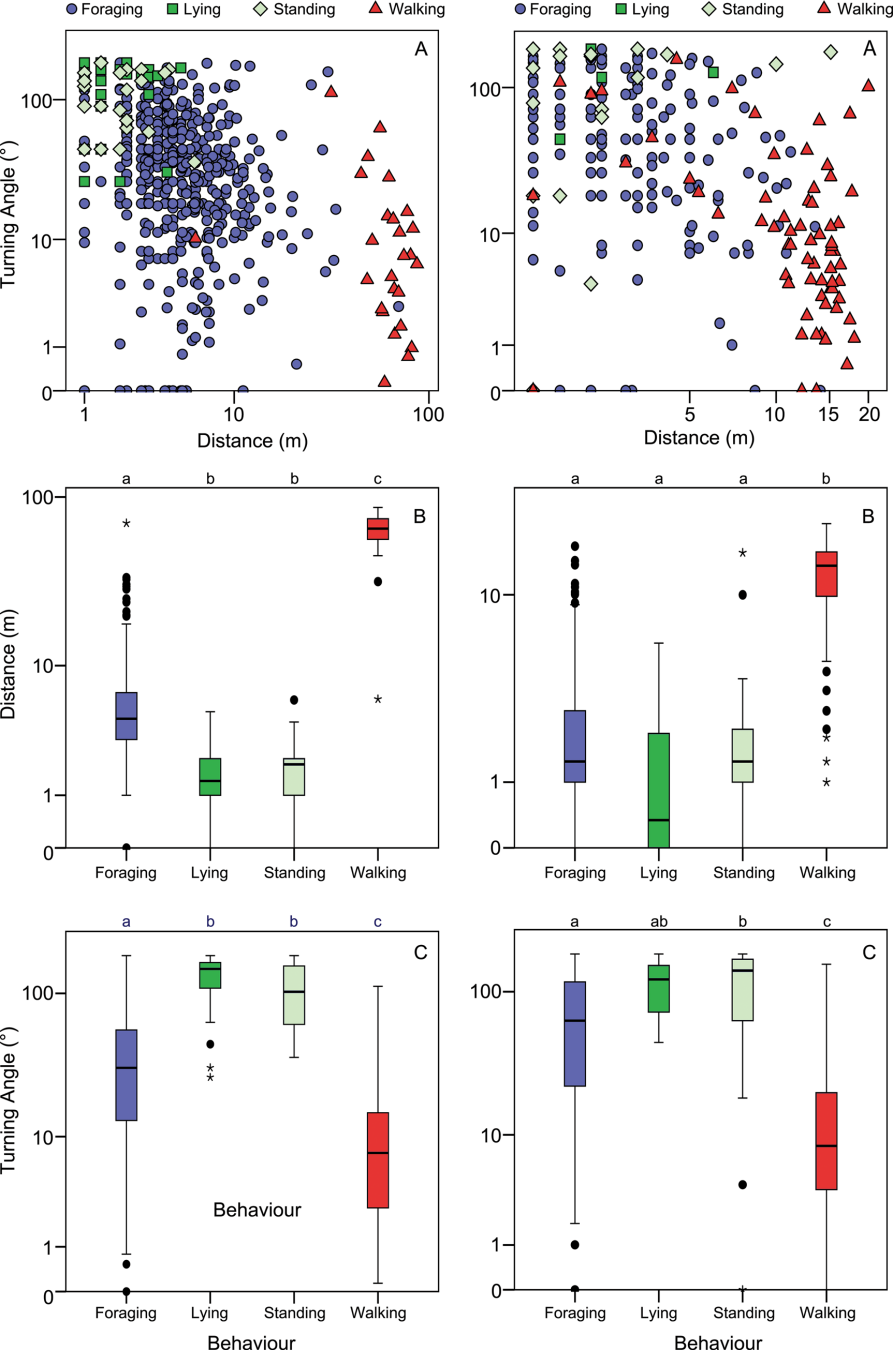
Turning angles and distances per behaviour type in the open field for the 1 min and 12 s interval. Distribution of turning angles and distances for the data from the 1 min (left) and 2 s (right) interval of cows in the open field for each of the four dominant types of behaviour. A) Relationship between turning angle and distance on a logarithmic scale. B, C) Boxplots showing the distribution of both distances and turning angles. Letters on top of the graphs depict if there are significant differences for these movement metrics among the groups (permutation ANOVA).

Based on the dataset of the 1 min interval from the open field Walking could be distinguished most clearly, because the distance covered between GPS fixes on a 1 min interval was much larger than for the other behavioural classes and the turning angle for Walking was relatively small (Fig 1). Foraging had the largest variation in both distances and turning angles. Lying and Standing were associated with small distances (Fig 1), but both these types of behaviour included a large variation in turning angles, even up to 180°. 64% of the Lying and Standing samples had a turning angle above 90°. Although the movement metrics of Lying and Standing were expected to be similar, due to the fact that in both cases the animal is not changing position, it should be noted that in general the turning angles are slightly smaller for Standing. The variability of Foraging caused overlap with all other behaviour types, but distances were larger than for Lying and Standing, and turning angles were mostly smaller. Both distance (permutation ANOVA, *F*_3, 603_ = 782.46, *p* < 0.0001) and turning angle (*F*_3, 593_ = 95.77, *p* < 0.0001) differed significantly between behaviour types except for Lying and Standing (Fig 1).

A decision tree was created using all four behaviour types (S1 Fig). After pruning of this decision tree, using the cross-validation procedure, the decision tree was able to correctly distinguish 93% of the behaviours in the training data set. Next, this decision tree was used as the model for further classification of the 1 min interval GPS data and validated with the other half of the dataset. This resulted in an overall correctly classified percentage of 86% (Table 2). The decision tree was quite extensive with a large depth and 27 terminal nodes. The confusion matrix showed, however, that during validation of the tree no more than 10% of GPS data were correctly classified as Standing, and that they were often misclassified as Foraging or Lying.

**Table 2:** Confusion matrix for the decision tree based on distance and turning angle, for the four dominant types of behaviour (open field, 1 min interval).

Results training sample						Results validation				
Observed	Predicted					Predicted				
	Foraging	Lying	Standing	Walking	Correct (%)	Foraging	Lying	Standing	Walking	Correct (%)
Foraging	498	7	0	1	98	443	19	5	3	94
Lying	8	41	0	0	84	21	33	1	0	60
Standing	12	10	4	0	15	26	12	4	0	10
Walking	2	0	0	24	92	0	0	0	42	100
Overall (%)	86	10	1	4	93	81	11	2	7	86

To the left the results of the training sample that was used for tree building and on the right the results after validation of the decision tree.

Distances and turning angles for Lying and Standing were not significantly different and these two types of behaviour could not be distinguished from each other based on the CART tree. Lying and Standing are similar types of behaviours for which no or only little displacement of the GPS signal was expected with subsequently no difference in movement metrics. It was therefore decided, as in other studies [12, 15], to pool Lying and Standing as one type of behaviour named ‘Resting’. Taking these two behaviours together as Resting had the advantage of being able to create a much smaller decision tree (S2 Fig, Table 3) with fewer decision rules and a higher overall percentage of data that was correctly classified during validation (88%).

**Table 3:** Confusion matrix for the decision tree based on distance and turning angle for the three types of dominant behaviour (open field, 1 minute interval) where Lying and Standing are taken together as Resting.

	Result training sample				Result validation			
Observerd	Predicted			Correct	Predicted			Correct
	Foraging	Resting	Walking		Foraging	Resting	Walking	
Foraging	488	17	1	96	432	35	3	92
Resting	16	59	0	79	36	61	0	63
Walking	2	0	24	92	0	0	42	100
Overall (%)	83	13	4	94	77	16	7	88

To the left the results of the training sample that was used for tree building and on the right results after validation of the decision tree.

We also ran the CART tree (S1 Fig) for all 1 min intervals during which multiple behaviour types were recorded, with the aim to classify these intervals as the behaviour that was most dominant within these intervals. With the use of the decision rules based on distance and turning angle from the CART tree with four behaviour types, the intervals with mixed behaviour were correctly classified as the dominant behaviour for 72% of the cases during validation (Table 4). However, only Foraging had a high percentage (96%) of correctly classified intervals.

**Table 4:** Confusion matrix for the data with mixed behaviour between GPS fixes (open field, 1 minute interval), to be classified as the most dominant behaviour within each interval.

Observed	Predicted				Correct (%)
	Foraging	Lying	Standing	Walking	
Foraging	831	16	3	18	96
Lying	18	8	0	0	31
Standing	124	18	5	7	3
Walking	172	5	0	126	42
Overall (%)	85	4	1	11	72

Classification was based on the decision rules from the CART tree as shown in S1 Fig.

#### 12 s interval

For the 12 s intervals based on the GPS data from the open field, distinguishing the behaviour types based on differences in distances and turning angles was relatively difficult. Only Walking differed significantly from the other behaviour types in terms of distance (*F*_3, 495_ = 228.53, *p* < 0.0001) and turning angle also differed among behaviour types, mainly due to the smaller turning angles of Walking (*F*_3, 450_ = 19.05, *p* < 0.0001; Fig 1C). Similar to the 1 min data turning angles for Lying and Standing were relatively high, with 54% of the turning angles for Lying and Standing higher than 90°.

The decision tree for the 12 s interval failed to correctly classify the data that included Lying and Standing (Table 5). After validation of the decision tree, the intervals during which Walking and Foraging were observed were correctly classified in 78% and 96% of the cases respectively, but intervals containing Lying and Standing were all incorrectly classified as Foraging.

**Table 5:** Confusion matrix for the decision tree based on distance and turning angle for the four dominant types of behaviour in the 12-second interval (open field).

	Result training sample					Result validation				
Observed	Predicted				Correct (%)	Predicted				Correct (%)
	Foraging	Lying	Standing	Walking		Foraging	Lying	Standing	Walking	
Foraging	197	0	0	0	100	189	0	0	8	96
Lying	6	0	0	0	0	6	0	0	0	0
Standing	8	0	2	0	20	11	0	0	0	0
Walking	5	0	0	31	86	8	0	0	28	78
Overall (%)	87	0	1	12	92	86	0	0	14	87

To the left the results of the training sample that was used for tree building and on the right the results after validation of the decision tree.

#### 2 s interval

Analysis of the movement metrics from the open field data at a 2 s interval showed that there were significant differences between behaviour types for distance (permutation ANOVA; *F*_3, 1185_ = 163.20, *p* < 0.0001). Nevertheless, Lying did not differ significantly in terms of distance from both Standing and Foraging. Moreover, turning angle did not differ among behaviour types (*F*_3, 1185_ = 1.85, *p* = 0.127). The turning angles for Lying and Standing were on average relatively low compared to the 1 min interval. 12% of the Lying and Standing samples had a turning angle above 90°. The distribution of distances and turning angles showed even more overlap between the different types of behaviour than in the 12 s data, and therefore it was not possible to build a decision tree that could distinguish the different types of behaviour based on distances and turning angles at this interval.

### Forest

In the forest, all sensors had a 100% fix success rate. The average positional accuracy of the sensors inside the forest and outside of the forest was 6.0 and 1.7 m, respectively. For the 1 min, 12 s and 2 s time interval in the forest, respectively 2250, 712 and 1273 intervals between two subsequent fixes could be linked to simultaneous observations of which 1455, 619 and 1239 intervals contained only one type of behaviour.

#### 1 min interval

**Fig 2:**
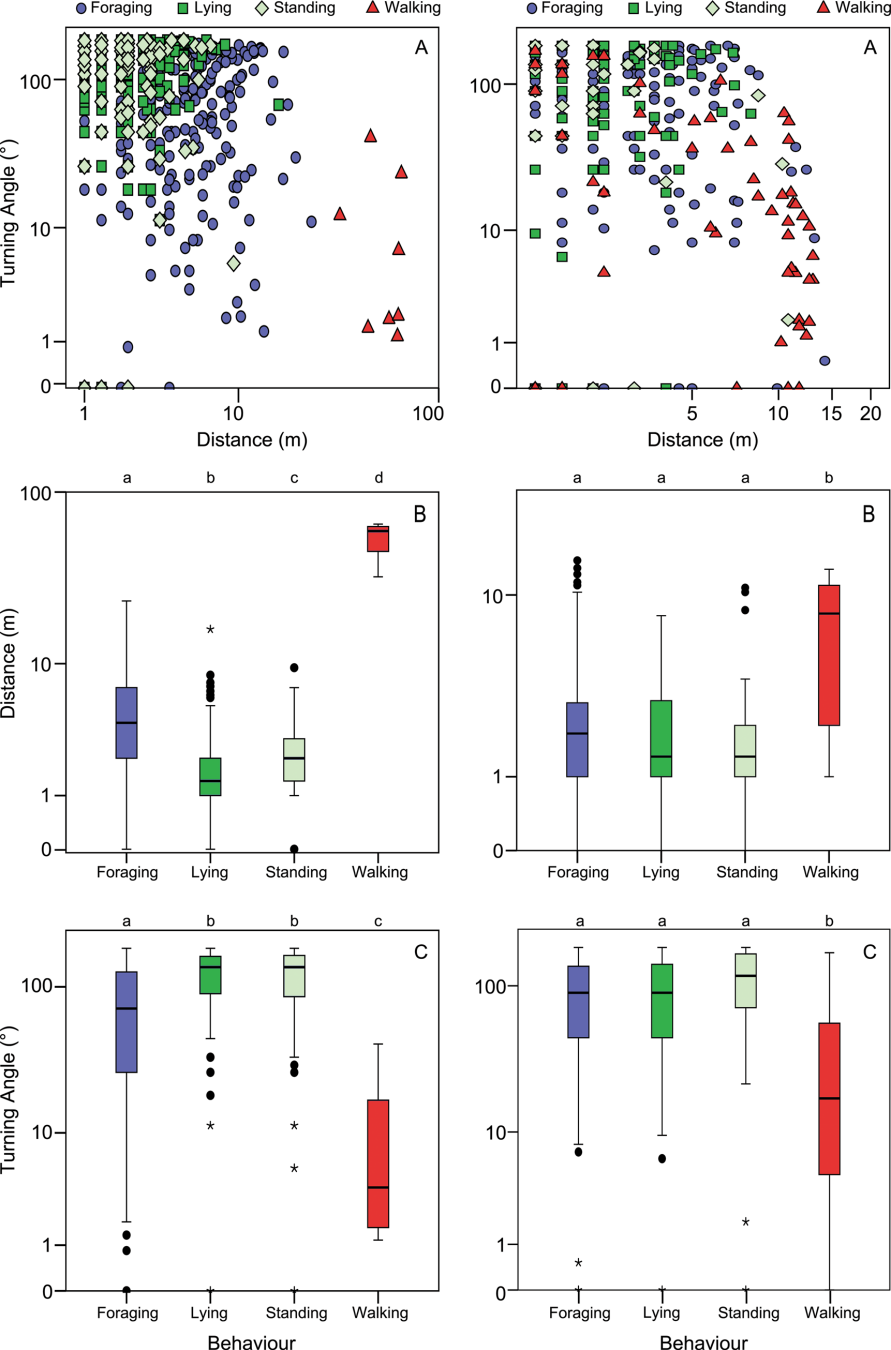
Turning angles and distances per behaviour type in the forest for the 1 min and 12 s interval. Distribution of turning angles and distances for the data from the 1 min (left) and 2 s (right) interval of cows in the forest for each of the four dominant types of behaviour. A) Relationship between distance and turning angle on a logarithmic scale. B, C) Boxplots showing the distribution of both distances and turning angles. Letters on top of the graphs depict if there are significant differences for these movement metrics among the groups (permutation ANOVA).

For the 1 min interval data from the forested area, significant differences were found between distances (*F*_3, 1435_ = 708.50, *p* < 0.0001; Fig 2) and turning angles (*F*_3, 1353_ = 66.22, *p* < 0.0001) for almost all behaviour types. However, Lying and Standing could not be statistically distinguished in terms of angles. Similar to the data from the open field these behaviour types were both described by very high turning angles, with 54% of the turning angles above 90°.

The overall percentage of correctly classified data using the CART tree (57%) was lower than for the data of the cows in the open field. Intervals that included Standing were never correctly classified, and were incorrectly classified as either Foraging or Lying. A second decision tree, created with three behaviour types (Lying and Standing taken together as Resting) improved the percentage of correctly classified data to 63%, but Resting data was often misclassified as Foraging, and vice versa (Table 6).

**Table 6:** Confusion matrix for the decision tree based on distance and turning angle for three dominant types of behaviour (forest, 1 min interval) with Lying and Standing taken together as Resting.

	Result training sample				Result validation			
Observed	Predicted			Correct (%)	Predicted			Correct (%)
	Foraging	Resting	Walking		Foraging	Resting	Walking	
Foraging	177	99	0	64	117	157	2	42
Resting	73	499	0	87	54	244	0	82
Walking	0	0	8	100	1	0	8	89
Overall (%)	29	70	1	80	30	69	2	63

To the left the results of the training sample that was used for tree building and to the right the results after validation of the decision tree.

#### 12 s interval

The 12 s interval data from the forest showed a similar pattern as the 12 s interval data from the open field, with significant differences between distances (*F*_3, 535_ = 16.11, *p* < 0.0001) and turning angles (*F*_3, 535_ = 78.58, *p* < 0.0001) for Walking compared to the other behaviour types (Fig 2). The decision tree that was created could correctly classify the intervals during which Walking and Foraging were observed for 42% and 97% of the cases respectively, but intervals containing Lying and Standing were almost all incorrectly classified as Foraging (Table 7).

**Table 7:** Confusion matrix for the decision tree based on distance and turning angle for the four dominant types of behaviour in the 12-second interval (forest).

	Result training sample					Result validation				
Observed	Predicted				Correct (%)	Predicted				Correct (%)
	Foraging	Lying	Standing	Walking		Foraging	Lying	Standing	Walking	
Foraging	173	0	0	1	99	171	0	0	5	97
Lying	69	0	0	0	0	70	0	0	0	0
Standing	28	0	0	0	0	27	0	0	2	0
Walking	14	0	0	13	48	15	0	0	11	42
Overall (%)	95	0	0	5	62	94	0	0	6	61

To the left the results of the training sample that was used for tree building and on the right the results after validation of the decision tree.

#### 2 s interval

Analysis of the 2 s interval data from the forest showed significant differences between behaviours for both distances (*F*_3, 1131_ = 38.43, *p* < 0.0001) and turning angles (*F*_3, 770_ = 32.08, *p* < 0.0001). For both movement metrics, Standing and Walking differed significantly from Foraging and Lying, but Standing and Walking were not significantly different from each other, as was the case for Foraging and Lying. In addition, for all behaviour types distances moved were smaller (< 3 m in 2 s) than the average positional accuracy of the sensors inside the forest (6.0 m, see above). Turning angles were relatively small for all behaviour types as well, with only 10% of the turning angles for Lying and Standing being higher than 90°. Therefore, based on these movement metrics, not one of the behaviour types was clearly distinguishable from the rest and as a result classification of the GPS data using a decision tree was unsuccessful.

## Discussion

This study shows that simple movement metrics derived from high-frequency GPS locations can be used to correctly infer behaviour from GPS data. Although in both the open field and the forest study only a small group of cows was fitted with tracking sensors, the data from the open field and the forest show the same pattern in terms of the relationship between distance and turning angle for each type of behaviour. This shows that, despite the influence of habitat type and differences in the accuracy of the GPS signal, the relationship between movement metrics and behaviour is able to describe the behaviour of cows in general. Also the time expenditure was often the same, as most cows ruminated or foraged at the same moment in time. In addition Mitlöhner et al. [37] found that during focal animal sampling, one cow was already representative of an entire pen of 10 animals. The data are therefore found to be representative of the larger population present.

Other studies already suggested how movement metrics can be used to deduce the behaviour of an animal [10, 11, 38, 39]. However, many studies do not account for the fact that the theoretical relationship between behaviour and movement metrics is different from the link between behaviour and movement metrics derived from GPS data. In this study, Lying and Standing were never distinguished by their relatively small distances in combination with small turning angles, in contrast to the predictions from Guo et al. [12]. Even while the animal is not moving, there is always a positional error (however small) of the GPS location [40]. The GPS signal seems to ‘jump’ back and forth around the true location, which can results in turning angles close to 180° for those situations [41]. This effect was less prominent at smaller time intervals, which might be caused by the application of smoothing algorithms (usually Kalman filters) that were present in the GPS sensors. In general, the smoothed data are more accurate, but the position of a data point becomes dependent on the previous locations with the application of the algorithm [9]. This dependency is higher at smaller time intervals and therefore the smoothing effect is larger, probably explaining the lower frequency of turning angles close to 180° at smaller time intervals.

Hurford [41] stresses that these high turning angles should be treated with suspicion and that filtering, in order to remove high turning angles that occur during a small displacement, may remove the data that is not biologically meaningful. Adams et al. [42] excluded for example all data with turning angles above 160° from their analysis. However, it is possible that such large turning angles are associated with behaviour, like searching behaviour while foraging. Applying data correction or filtering might then also remove valuable information with regard to movement patterns that does in fact correctly render the behaviour of an animal. Moreover, if in this study the high turning angles were to be removed, even more overlap in movement metrics would be found between Foraging and Standing and Lying, because these large turning angles mainly occur in stationary behaviour only. Hurford [41] also pointed out that, in the same way as decreased fix success rate, filtering can strongly reduce the number of movement metrics (turning angles or distances) that can be calculated. We therefore did not apply a filtering technique to our analysis.

CART decision trees proved to be a relatively simple and an efficient tool for the classification of this type of movement data. We were able to make decision trees with robust classification criteria, independent of the noise in the data [33–36], and showed how these can be applied to new datasets (e.g., the intervals with a mixture of behaviours) with the same variables [3, 14, 16]. CART trees are less computationally intensive and they stand out by intelligibility compared to other classification methods like Random Forests or Neural Networks, which were suggested by Nathan et al. [16] as a method to obtain a higher accuracy. Classification was most successful for the 1 min time interval data from the open field. Based on distance and turning angles, more than 70% of the intervals between fixes could be correctly classified using a CART decision tree.

Our CART decision tree was initially trained based on intervals with only a single type of behaviour. However, in reality it is not always known which and how many types of behaviour an animal performs between two fixes. Therefore all 1 min intervals during which multiple behaviour types were recorded were classified, with the aim to classify these intervals as the behaviour that was most dominant within these intervals. This showed that, not surprisingly, intervals that contain a combination of different behaviours are not as likely to be classified correctly. Although Foraging is classified quite well, the other types of behaviour are often misclassified as Foraging. Foraging behaviour is circumscribed by a large and varying range of turning angles and distances [10, 11] and it is therefore very likely that combinations of behaviours within an interval are often misclassified as Foraging. Hence, when Lying or Standing takes place in the same interval as Walking, the intervals are mostly misclassified as Foraging.

Turchin [38] stated that behaviour can be best described by GPS data when the time interval is similar to the duration of the behaviour. Using an interval smaller than 1 min could have the advantage to better capture behaviours that often do not last one full minute. In contrast to what was expected, shortening the time interval between GPS fixes did not improve classification. Movement metrics calculated with the data from the 2 s intervals turned out not to be distinctive for the different types of behaviour. The exploratory tests showed that the sensors had an average positional error of 2.1 m. This shows that the time interval should also be long enough for the behaviour to define itself by its characteristic movement metrics, and GPS positions should be sufficiently accurate to distinguish stationary behaviour from Foraging at such a high sampling frequency. At a 12 s interval the classification improved, but there was still a large overlap in movement metrics among behavioural classes. Ungar et al. [14] suggested that fix intervals larger than 1 min might have the advantage to distinguish Foraging better from Lying and Standing, because the distance covered by Foraging is then expected to differ more from the location error that is present when the animal is not moving. It might therefore be interesting to apply the classification method for a whole range of time intervals to obtain the optimum time interval at which location data can be best classified as the correct behaviour. This could not be investigated further in the current study, due to limitations in sample size. The optimal time interval will however be dependent on the type of behaviour and the species. Moreover, the duration and scale of the behaviour may depend on the size and body mass of the animal, with larger animals displaying fewer types of behaviour within the same time interval than smaller animals [43]. Therefore, further research is recommended on the use of suitable time intervals for further innovation of tracking studies.

As was expected based on the decrease in GPS performance under forest cover [22–26], we found that the data from the forested area could not be classified as well as the data from the open field. However, this was probably not only caused by the larger positional error (6.0 m in the forest versus 2.3 m in the open field), but also influenced by habitat type. The cows in this forested area not only grazed (as in the open field study), but also browsed. During browsing a cow often remained longer in one place, while eating the leaves of a tree, than during grazing. The relatively larger error of the GPS locations, in combination with the fact that browsing was recorded as Foraging, created an overlap between the location data in terms of movement metrics, and this could be the reason for the larger classification errors [20, 29]. This illustrates that knowledge on the behaviour of an animal in relation to its habitat is very important in order to understand the relation between automatically retrieved data and the behaviours a researcher defines [10, 29]. In addition, factors like weather conditions, sex and age could influence the performance of behaviour and therefore the related movement metrics [44]. Previous studies concluded that a higher temporal resolution might work better in forests because it counteracts the smaller fix success rate that is obtained under forest cover [26]. However, our study shows that with improved tracking systems, a high (100%) fix success rate can be obtained. Positional accuracy then has the most prominent effect on the tracking data, leading to the conclusion that in a forested habitat a higher temporal resolution may be less suitable for inferring behaviours because of the poor distinction between true movements and the larger positional error.

The results from this study stress the importance of understanding the relationship between behaviour and movement metrics derived from GNSS data and show how this is affected by the use of different time intervals in different habitats. Due to the increasing amount of available wildlife tracking data with an increasing spatiotemporal resolution, it becomes possible for researchers to establish classification rules suitable for their tracking data. The lower costs of the GPS devices, and smaller weight of these devices enable a wider application. In wildlife studies it is now possible to identify calving behaviour in red deer, or the location of wolf kills, using certain classification rules [45, 46]. The classification method and results within this study are therefore widely applicable to support this use of GNSS data. However, calibration and validation of classification algorithms remain required, as algorithms developed for, for instance, cows cannot be used for non-related species, and changes in habitat can affect behaviour and movement metrics. We therefore made two separate classification algorithms, one for the open field, and one for the forest. Classification algorithms can be improved by incorporating additional variables in the analysis, like changes in heading, turning angle distribution [11] or net displacement [14]. Moreover, the additional application of tri-axial accelerometers in GPS tracking devices can increase the behavioural classification accuracy [3, 16, 47, 48]. However, the drawback of using these additional variables is increasing complexity, and further demands on battery and memory capacity. The method used in our study (based on distances and turning angles) is easy to interpret and requires only single intervals for the calculations. Apart from wildlife research, the method used in this study may also prove to be useful for farmers in order to monitor for welfare and productivity of cows. Classification rules can be established and validated easily based on visual observations. Accurately inferring animal behaviour and the spatial heterogeneity in behavioural patterns, such as the foraging activity of animals, is possible from high resolution GNSS locations. In addition, individual deviations in behaviour of livestock might be detected, for instance when an animal is in oestrus, or affected by disease or predation [20].

In conclusion, analysis of high-frequency GNSS fixes can be used to infer the type of behaviour an animal is performing. Obviously, knowledge about the types of behaviour a species might perform is required. The continuing development of GNSS tracking devices, with an increasing spatiotemporal resolution, will facilitate the availability of data that is suitable to study animal movements on different scales.

## Supporting Information

S1 File Detail of sampling locations. Additional information on the habitat types at the sampling locations during the open field and the forest study.
(DOCX)

S2 File ‘Time of day’ as auxiliary variable. Supplementary analysis on the use of time classes as extra predictor variable.
(DOCX)

S1 Fig CART tree for the 1 min interval data from the open field for with all four dominant types of behaviour. CART tree depicting the decision rules based on distances and turning angles of cows for the classification of Foraging, Lying, Standing and Walking. At each level a leftward move indicates that the answer to the decision rule is true/yes, whereas a rightward move indicates the answer to the decision rule is false/no.
(TIF)

S2 Fig CART tree for the 1 min interval data from the open field for three dominant types of behaviour. CART tree depicting the decision rules based on distances and turning angles of cows for the classification of Foraging, Walking and Resting (Lying and Standing pooled into Resting). At each level a leftward move indicates that the answer to the decision rule is true/yes, whereas a rightward move indicates the answer to the decision rule is false/no.
(TIF)

S1 Dataset GPS and observation data open field. (XLSX)

S2 Dataset GPS and observation data forest. (XLSX)
