# Supplementary material for: Deriving Animal Behaviour from High-Frequency GPS: Tracking Cows in Open and Forested Habitat
Source: PLoS One. 2015 Jun 24;10(6):e0129030. doi: 10.1371/journal.pone.0129030 (PMC4479590; doi:10.1371/journal.pone.0129030)
Supplement: S1 File — Additional information on the habitat types at the sampling locations during the open field and the forest study. (DOCX) [file pone.0129030.s001.docx]

**S1 Supporting information: Detail of sampling locations**

The open field study was performed on a farm with dairy cattle near Wageningen, the Netherlands (52°01’ N, 5°62’ E). The cows grazed in an open field of 3.2 ha adjacent to the farm between 08:00 and 17:00 hours. A watering point was attached to the stable, where the cows could drink freely during the day (Fig. 1). The field was split into two parts in longitudinal direction by a barbed wire fence.


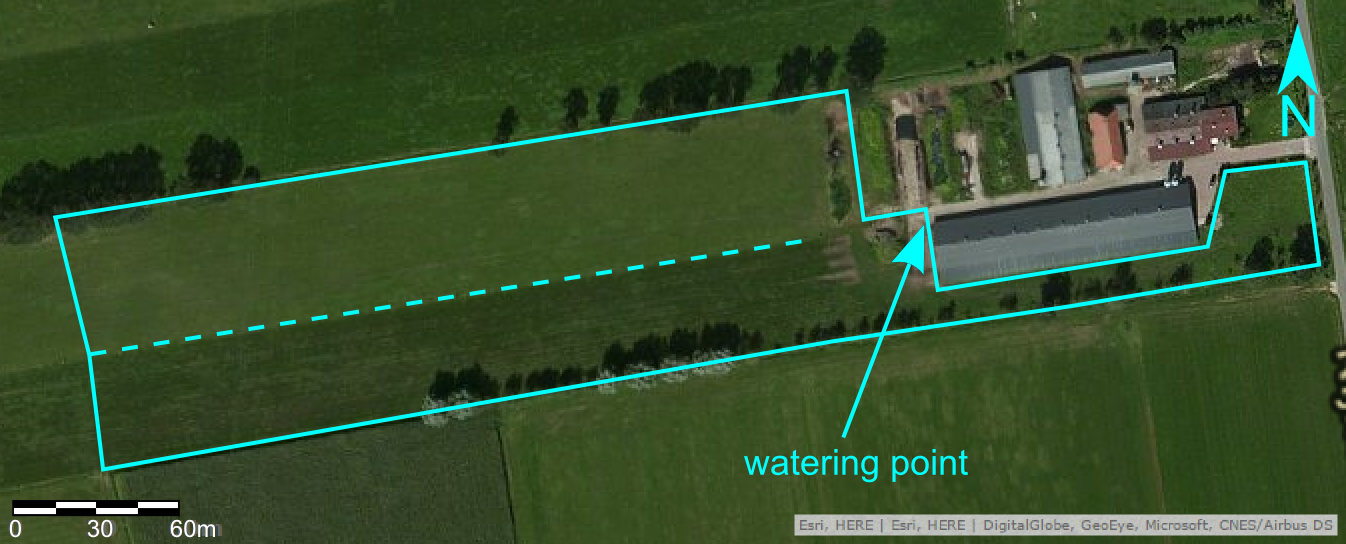

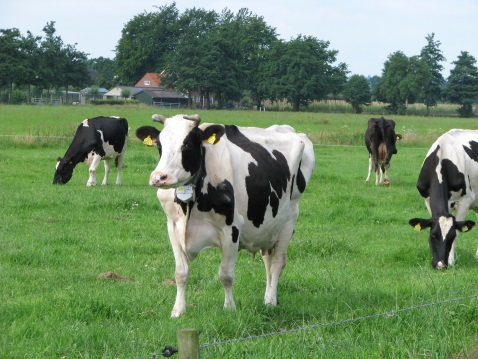
**Figure 1. Sampling in the open field with the outline of the study site (left) and example of field conditions (right). The dotted line within the outline indicates the barbed wire fence.**

The forest study was performed at the “Renkumse Heide” near Wageningen (52°01’ N, 5°75’ E), where eight free-ranging cows were fitted with GPS tracking receivers. The cows could roam freely in a forest of 47.0 ha during the whole day. Observations were carried out in the morning and the afternoon, similar to the open field study. Alongside one of the borders of the forest there was a small strip of land where the cows could walk to access a watering point (Fig. 2). The forest consisted of mixed woodland (deciduous and coniferous trees) with an understory of small shrubs and trees.


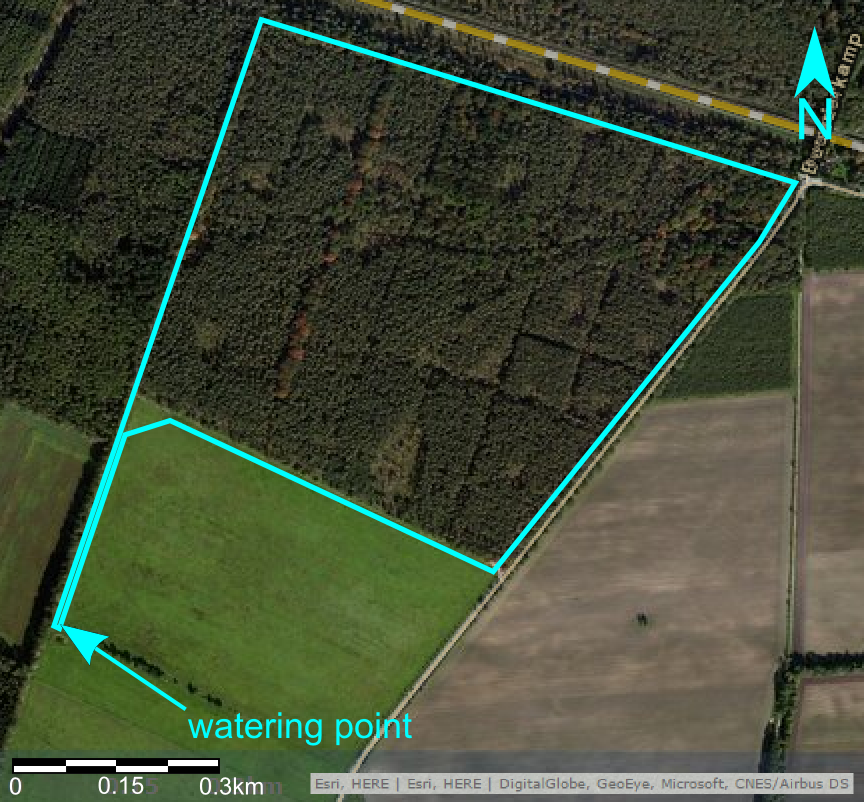

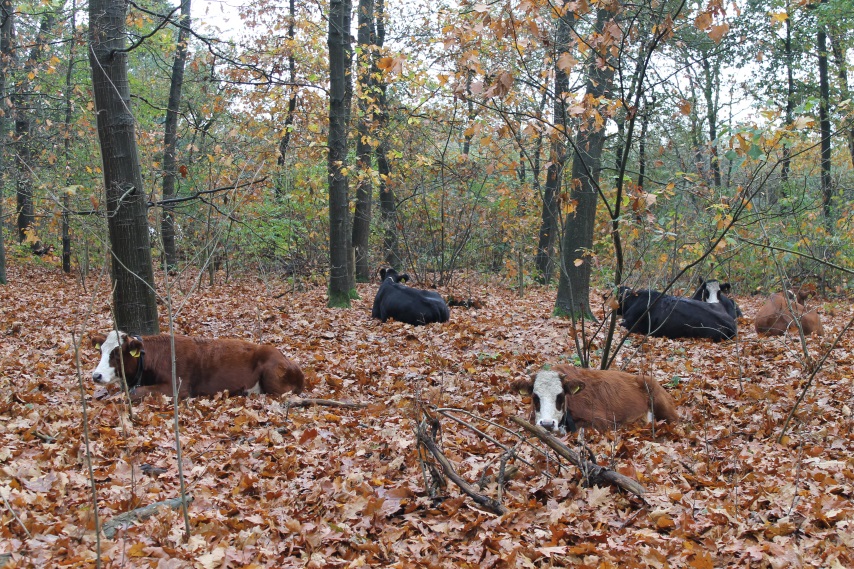
 **Figure 2. Sampling in the forest with the outline of the study site (left) and example of field conditions (right).**
