# Supplementary material for: Deriving Animal Behaviour from High-Frequency GPS: Tracking Cows in Open and Forested Habitat
Source: PLoS One. 2015 Jun 24;10(6):e0129030. doi: 10.1371/journal.pone.0129030 (PMC4479590; doi:10.1371/journal.pone.0129030)
Supplement: S2 File — Supplementary analysis on the use of time classes as extra predictor variable. (DOCX) [file pone.0129030.s002.docx]

**S2 Supporting information – ‘Time of day’ as an auxiliary variable**

**Introduction**

Cows (and other animals) often show clear activity patterns in the course of a day. For example, activity patterns of cows arise due to the alternation between foraging and ruminating. From this, we hypothesized that the time at which a particular behaviour occurred, can be used as an extra predictor variable, improving the correct classification of the GPS data [1-3].

**Methods**

In order to explore the use of ‘Time of day’ as an auxiliary variable, the activity pattern of our cows in the open field study was analyzed. The time of day variable was constructed by classifying all observation data with regard to the hour of observation, i.e. 6:00h (from 6:00-7:00h), 7:00h (7:00-8:00h) etc. A second classification was made using half hour time intervals. This resulted in 8 and 15 time bouts, respectively. A total of 358 minutes (intervals) of observation data were available from 06:00h to 14:00h. The percentage of time allocated to the four dominant behaviours was calculated in each minute of observation. Next, apart from distance and turning angle, the time of day in which every 1 min GPS location sample occurred, were assigned to the data. Subsequently, various decision trees were created by adding the time of day as an extra variable to the tree building algorithm (next to distance and turning angle). The resulting decision rules were therefore based on three variables, instead of two.

**Results**

The analysis based on time of day, using one hour intervals, revealed no clear pattern in foraging behaviour, as during the entire observation period from 6:00-14:00h foraging was always the dominant behaviour (Figure 1). When the analysis was repeated using half an hour intervals, a few peaks were visible during which Standing and Lying were the more dominant behaviour. Lying and Standing were observed more often later during the day, which suggests that at that time of the day the cows spent more time Lying. In contrast, the first hour after the cows were released into the field, the cows were only Foraging and Walking, whereas Standing and Lying were never observed.

The best decision tree was obtained by incorporating the one hour time intervals, as data recorded when the animals were Standing was slightly less often misclassified as Lying (Table 1), compared to when only distance and turning angle were used. However, the data during which the cows were Lying was often incorrectly classified as Standing when the one hour time intervals were used. Although the addition of time of day in one hour classes delivered a somewhat simpler decision tree (20 terminal nodes) than when using distances and angles only. However, the addition of time of day did not result in a higher overall percentage of correctly classified GPS data.


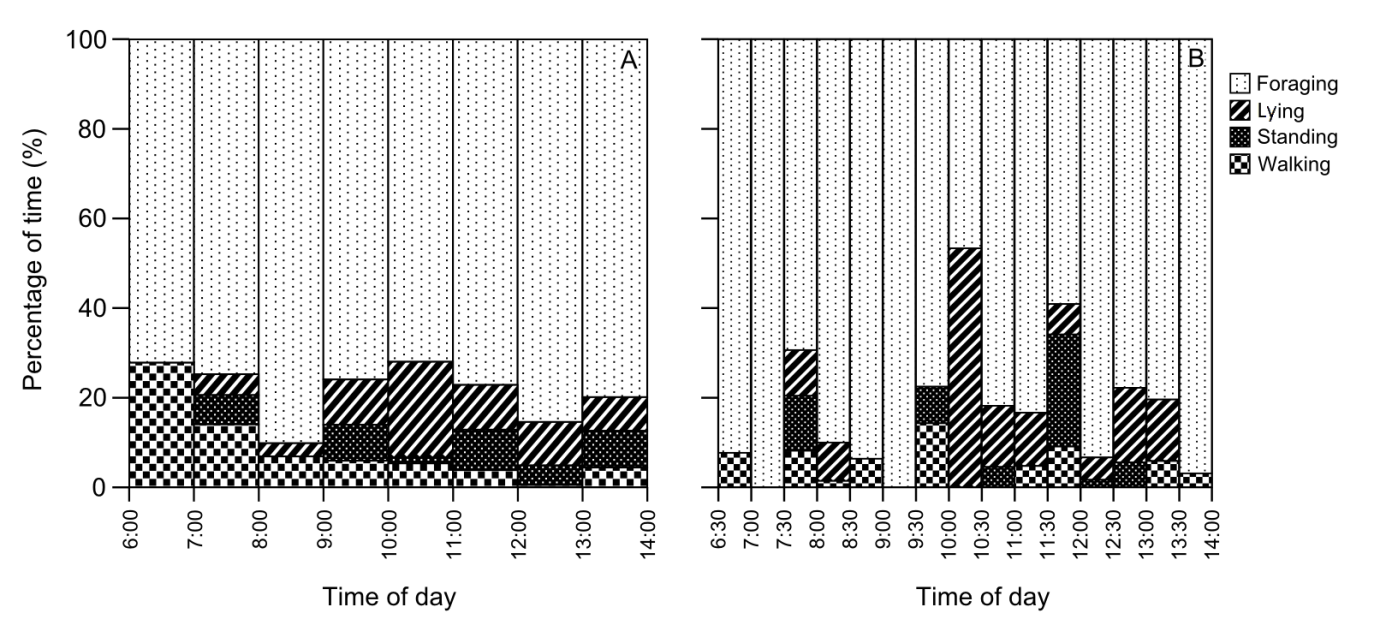


**Figure 1. Cumulative percentage of time spent over the complete 10 day observation period (open field) on each of the four dominant types of behaviour over time intervals of one hour (left) and half an hour (right), based on visual observations.**

**Table 1. Confusion matrix for the decision tree based on distance, turning angle, and time in intervals of one hour for the four dominant types of behaviour (open field, 1 minute interval). On the left the results of the training sample that was used for tree building and on the right the results after validation of the decision tree.**

| **Result training sample** | | | | | |  | **Result validation** | | | | |
| --- | --- | --- | --- | --- | --- | --- | --- | --- | --- | --- | --- |
| **Observed** | **Predicted** | | | | **Correct (%)** |  | **Predicted** | | | | **Correct (%)** |
|  | **Foraging** | **Lying** | **Standing** | **Walking** |  |  | **Foraging** | **Lying** | **Standing** | **Walking** |  |
| Foraging | 495 | 9 | 1 | 1 | 98 |  | 435 | 12 | 20 | 3 | 93 |
| Lying | 6 | 40 | 3 | 0 | 82 |  | 20 | 23 | 12 | 0 | 42 |
| Standing | 5 | 6 | 15 | 0 | 58 |  | 25 | 10 | 7 | 0 | 17 |
| Walking | 2 | 0 | 0 | 24 | 92 |  | 0 | 0 | 0 | 42 | 100 |
| Overall (%) | 84 | 9 | 3 | 4 | 95 |  | 79 | 7 | 6 | 7 | 83 |

**Discussion**

The addition of time of day as an auxiliary variable did not improve the classification of the GPS data compared to using distances and turning angles as variables only. In the case of the open field study, behaviour patterns in the course of the day appeared to contain too much variation to form a useful addition to the classification. Most likely, the time spent on the four most dominant behaviours was dependent on the time the cows were released into the field in the morning (which differed slightly each morning) and also the supplementation of dry forage, which was given by the farmer. Yet, the use of time of day as an auxiliary variable resulted in a simpler decision tree with fewer classification rules. This was considered a positive effect, because a simple tree is likely to be more generally applicable than a complex tree. A decision tree that is more general is probably going to be more accurate for the larger population, and is less likely to overfit (i.e. fit in outliers and noise in the data). Although in this case the daily pattern of the cows in the open field study was not clear enough to form a useful addition to the correct classification, time of day as an extra variable might be useful when animals show a clearer behavioural pattern during the day. However, in order to investigate if time of day is a useful variable, further studies are necessary, as there was insufficient data in this study to draw a firm conclusion.

**References**

1. Patterson TA, Thomas L, Wilcox C, Ovaskainen O, Matthiopoulos J. State-space models of individual animal movement. Trends Ecol Evol. 2008; 23: 87-94.
2. Frair JL, Fieberg J, Hebblewhite M, Cagnacci F, DeCesare NJ, et al. Resolving issues of imprecise and habitat-biased locations in ecological analyses using GPS telemetry data. Philos Trans R Soc Lond B Biol Sci. 2010; 365: 2187-2200
3. Martin J, van Moorter B, Revilla E, Blanchard P, Dray S, et al. Reciprocal modulation of internal and external factors determines individual movements. J Anim Ecol. 2013; 82: 290-300.
