# Supplementary figures and images for: Deriving Animal Behaviour from High-Frequency GPS: Tracking Cows in Open and Forested Habitat
Source: PLoS One. 2015 Jun 24;10(6):e0129030. doi: 10.1371/journal.pone.0129030 (PMC4479590; doi:10.1371/journal.pone.0129030)

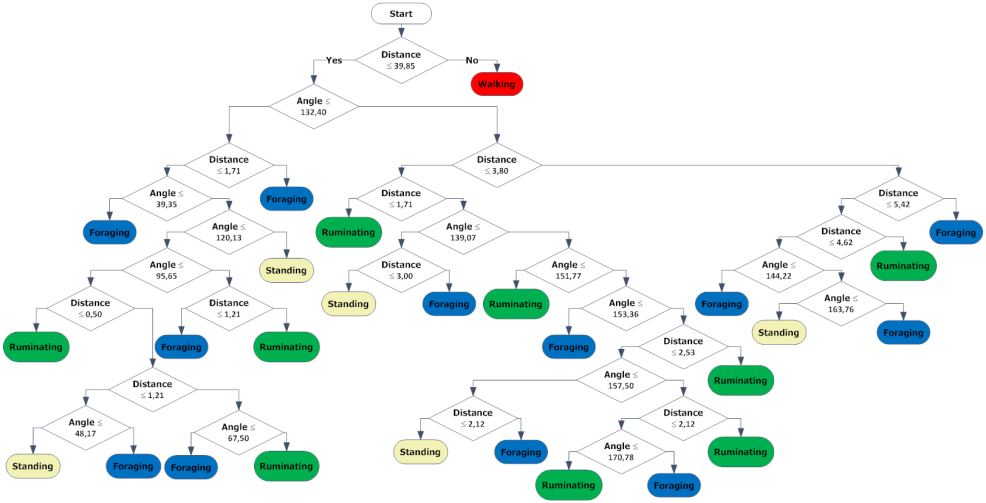

Supplement: S1 Fig — CART tree depicting the decision rules based on distances and turning angles of cows for the classification of Foraging, Lying, Standing and Walking. At each level a leftward move indicates that the answer to the decision rule is true/yes, whereas a rightward move indicates the answer to the decision rule is false/no. (TIF) [file pone.0129030.s003.tif]

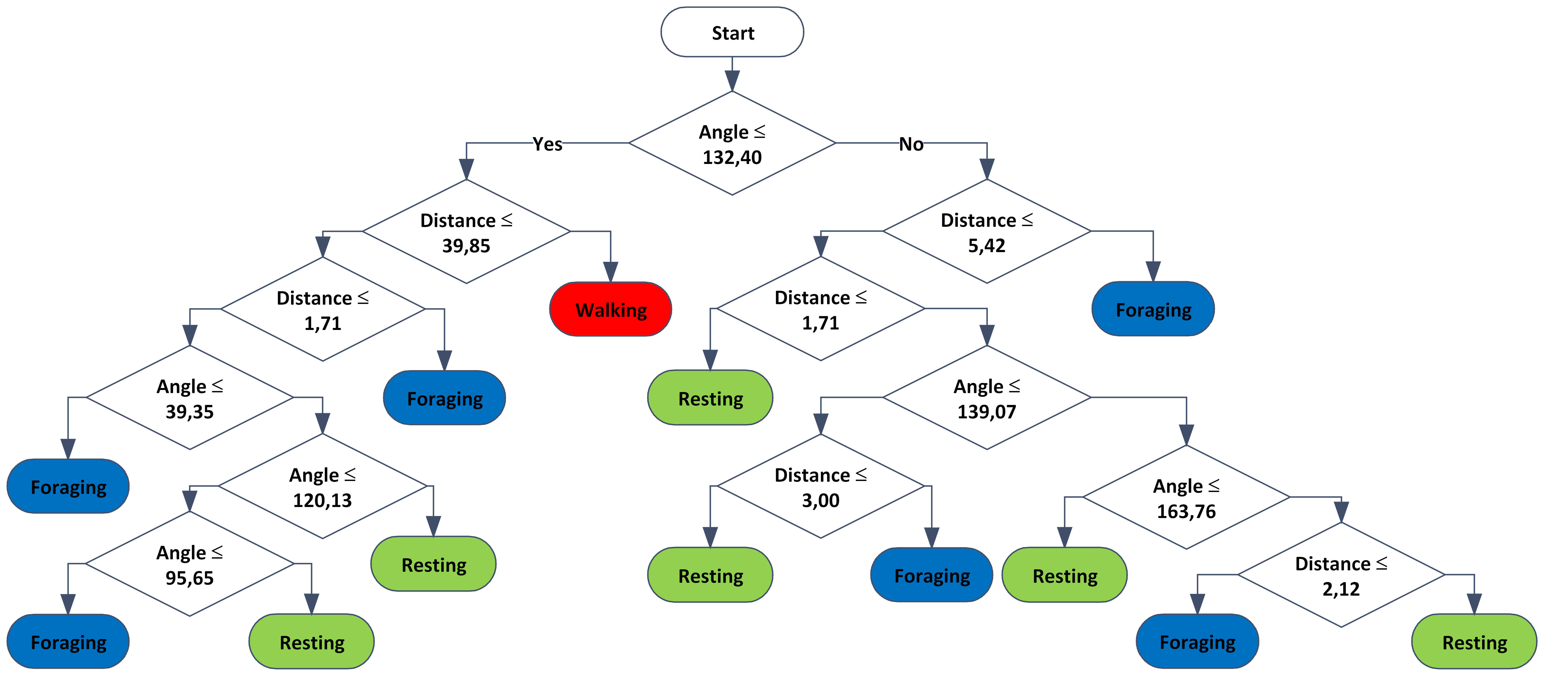

Supplement: S2 Fig — CART tree depicting the decision rules based on distances and turning angles of cows for the classification of Foraging, Walking and Resting (Lying and Standing pooled into Resting). At each level a leftward move indicates that the answer to the decision rule is true/yes, whereas a rightward move indicates the answer to the decision rule is false/no. (TIF) [file pone.0129030.s004.tif]
